# Georgia Medical Association

**Published:** 1873-03

**Authors:** 


Georgia Medical Association.—We learn that the Committee of Arrangements for the State Medical Association are making the most ample arrangements for entertaining the members of that body in a style not excelled upon any similar occasion in this State.

We can but hope that the contributions to science will eclipse what we are assured is intended to be a reception of the body upon the most elegant scale.

In behalf of the committee, and the profession here, we again most earnestly urge every regular medical man in the State to meet with us upon that occasion. We can entertain them all. We are informed by the Acting Secretary (Dr. J. T. Johnson) that the railroads have all agreed to an arrangement for half fare. We hear of quite a variety of plans on foot for making the occasion an interesting one, outside of the special objects for which the body assembles.

